# Supplementary material for: Females with obesity exhibit greater influenza vaccine-induced immunity and protection than males in a mouse model
Source: Front Immunol. 2025 Dec 18;16:1699275. doi: 10.3389/fimmu.2025.1699275 (PMC12756896; doi:10.3389/fimmu.2025.1699275)
Supplement: Supplementary file 1 [file Table1.docx]

Supplementary Material

**Supplementary Figure 1:**

**
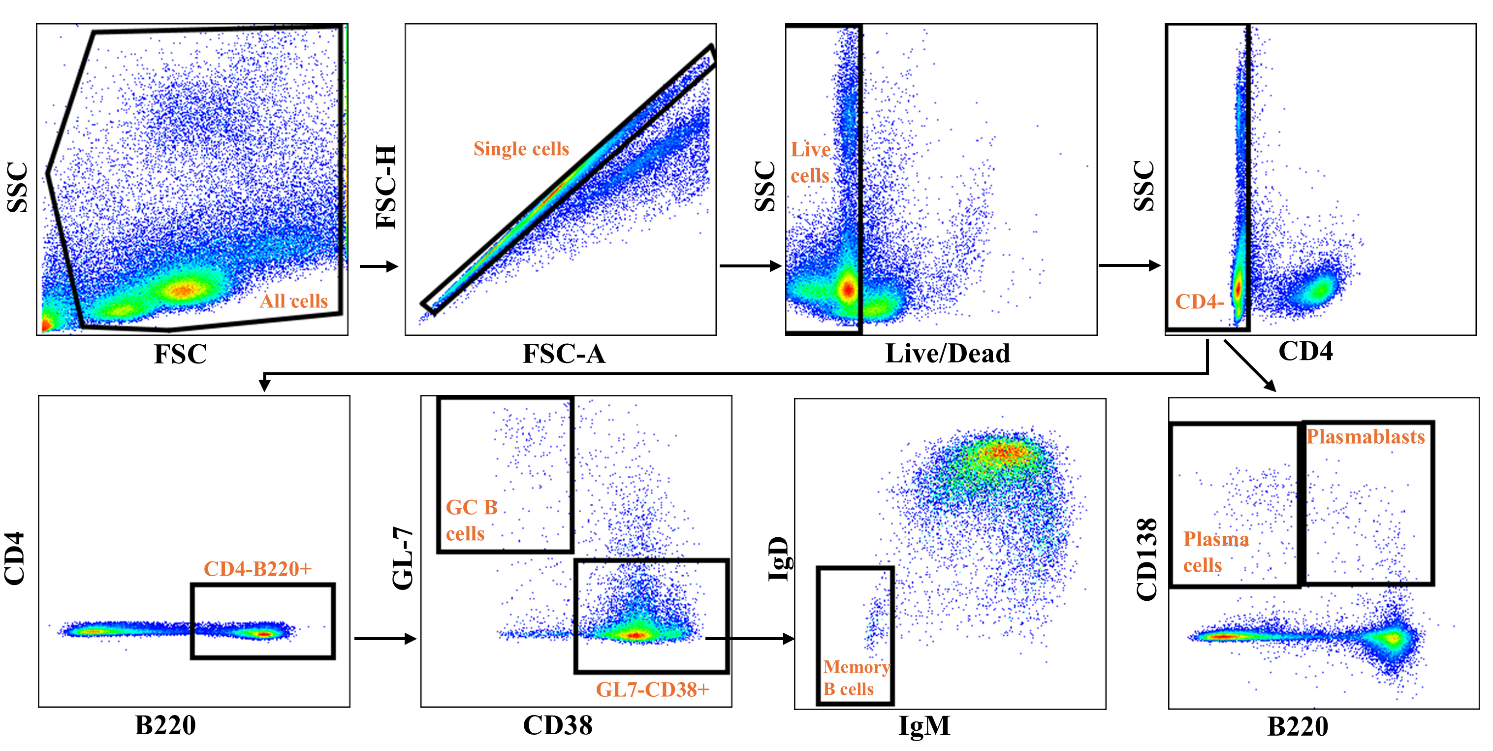
**

**Gating strategy for splenic B-cell subsets.** At 35 days post-vaccination, splenocytes were processed for flow cytometry, and a representative gating strategy is shown. It included: lymphocytes (FSC/SCC), single cells (FSC-H/FSC-A), live cells (viability dye negative), CD4^-^ (to exclude T cells), followed by plasmablasts (B220^+^CD138^+^) and plasma cells (B220^-^CD138^+^). From CD4^-^B220^+^ cells: germinal center (GC) B cells (CD38^-^GL7^+^) and memory B cells (GL7^-^CD38^+^IgD^-^IgM^-^) were gated.

**Supplementary Figure 2**

**
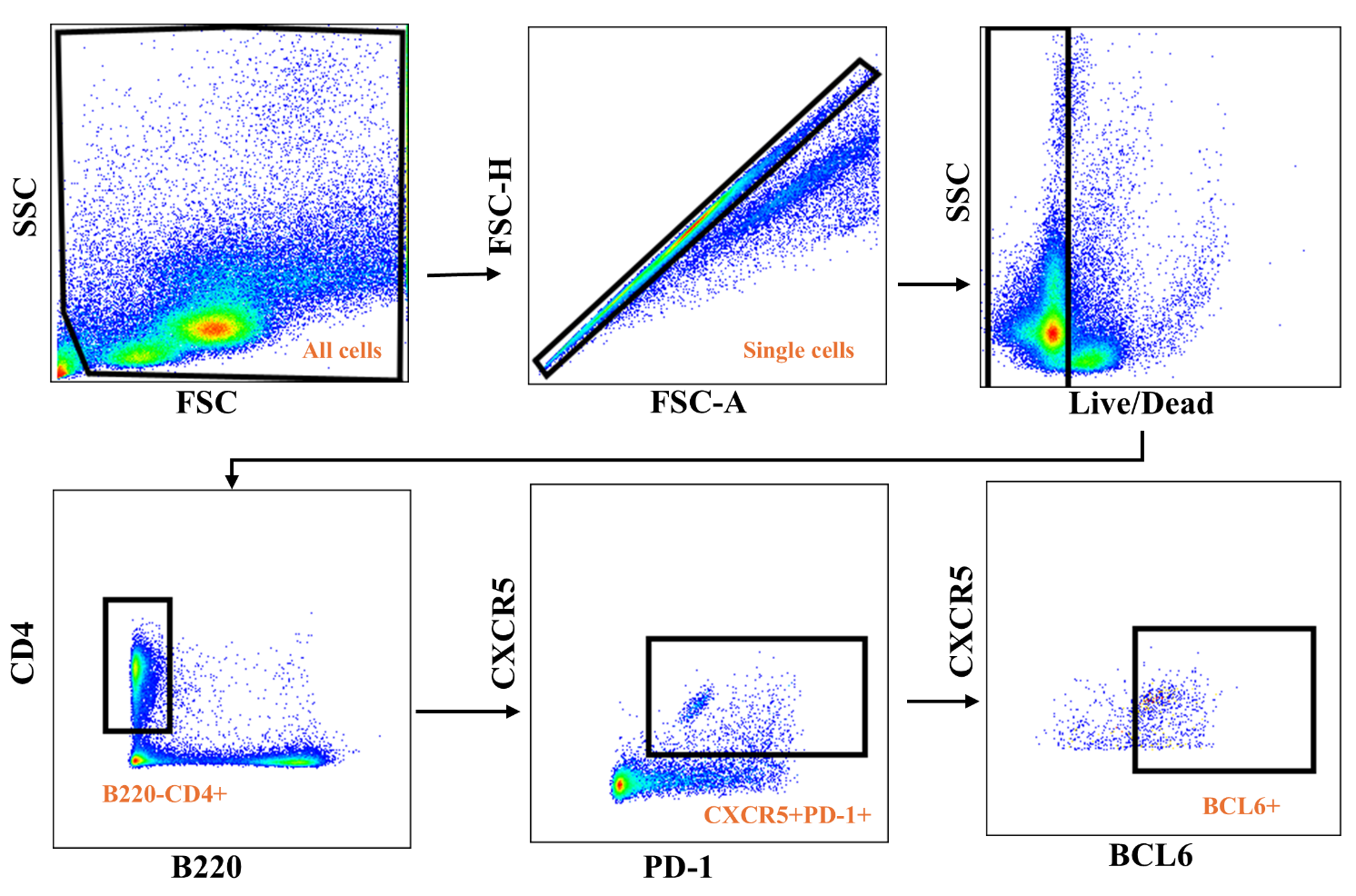
**

**Gating strategy for splenic T follicular helper (Tfh) cells.** Gating strategy included: lymphocytes (SSC/FSC), single cells (FSC-H/FSC-A), live cells (viability dye negative), CD4^+^B220^-^ (to exclude B cells), followed by gating for CXCR5^+^PD1^+^ cells and Tfh cells (CXCR5^+^PD-1^+^BCL6^+^).

**Supplementary Figure 3**

**
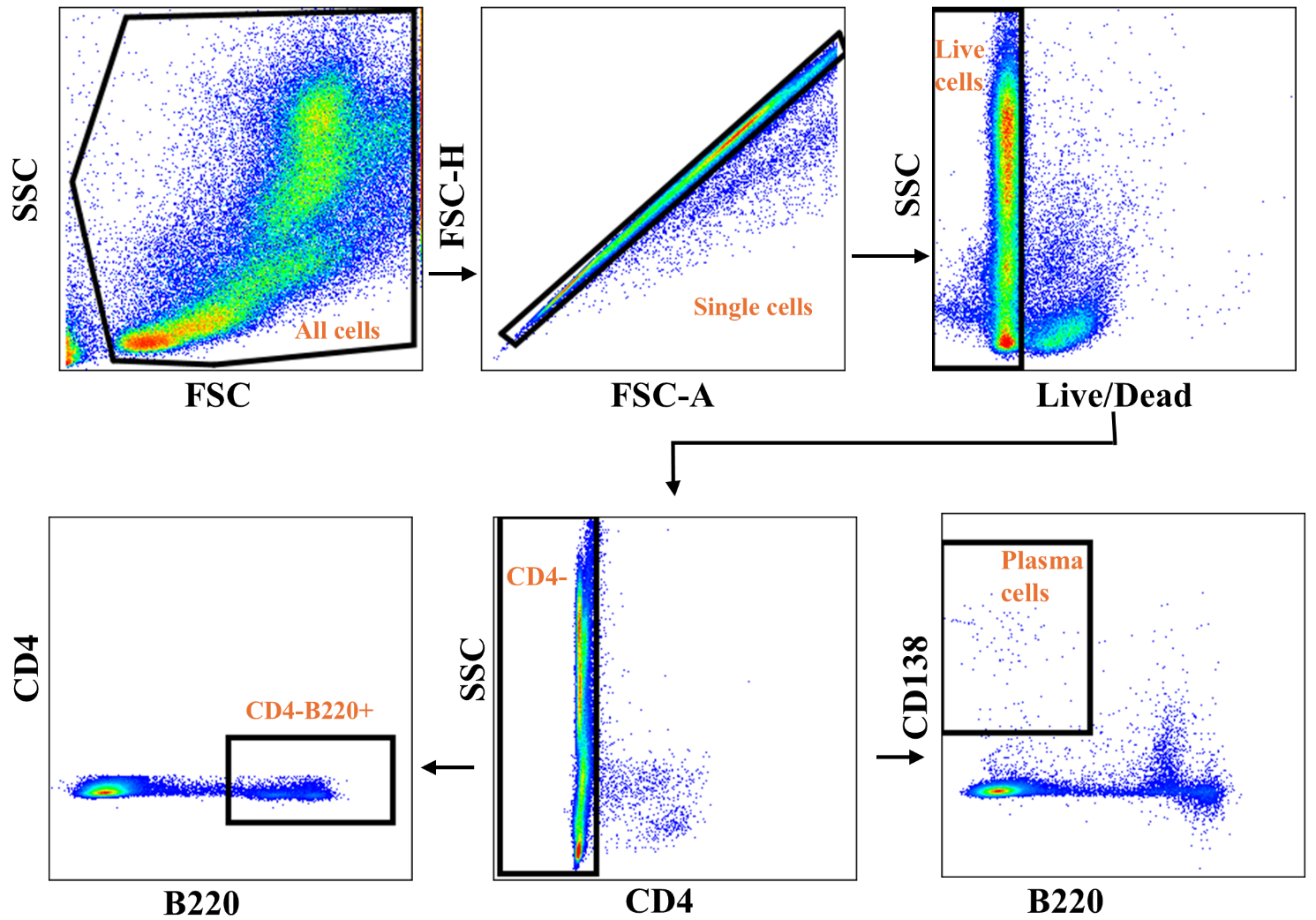
**

**Gating strategy for bone marrow plasma cells.** Gating strategy included: lymphocytes (SSC/FSC), single cells (FSC-H/FSC-A), live cells (viability dye negative), CD4^-^ (to exclude T cells), followed by B cells (CD4^-^B220^+^) or plasma cells (B220^-^CD138^+^).

**Supplementary Figure 4**

**
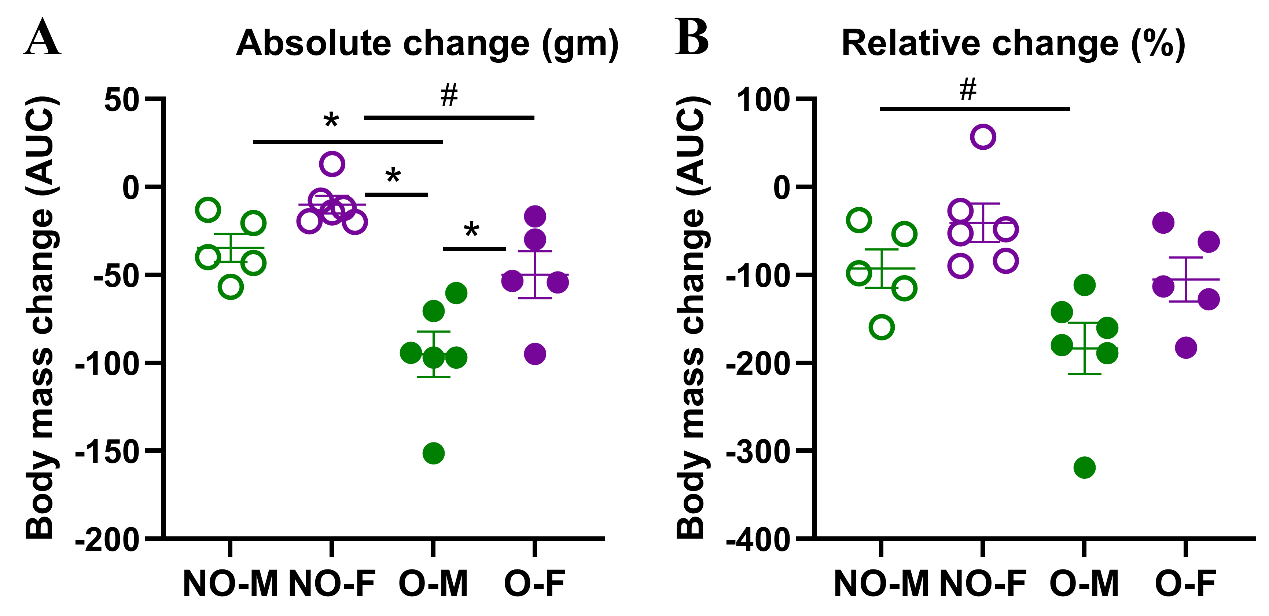
**

**Post-challenge absolute and relative body mass loss as area under the curve (AUC).** Vaccinated male and female mice, with or without obesity, were challenged with a drift variant of the 2009 H1N1 IAV at 42 days post-vaccination (dpv). Absolute body mass (g) and relative change (%) in body mass were determined and are presented in Figure 3A and 3B, respectively. In this figure, AUC values for (A) absolute body mass loss and (B) relative body mass loss are compared using two-way ANOVA followed by Tukey’s post-hoc analysis. An asterisk (*) indicates a significant difference (p<0.05) and a hash (^#^) represents a trend (0.05≤p≤0.1) between the groups. Abbreviations: NO-M: non-obese males; NO-F: non-obese females; O-M: males with obesity; and O-F: females with obesity.
